# Live Video Adaptations to a Mind-Body Activity Program for Chronic Pain and Cognitive Decline: Protocol for the Virtual Active Brains Study

**DOI:** 10.2196/25351

**Published:** 2021-01-04

**Authors:** Ryan A Mace, James D Doorley, Paula J Popok, Ana-Maria Vranceanu

**Affiliations:** 1 Integrated Brain Health Clinical and Research Program Massachusetts General Hospital Harvard Medical School Boston, MA United States

**Keywords:** chronic pain, cognitive decline, physical activity, mind-body therapies, aged, telemedicine, mobile phone

## Abstract

**Background:**

Chronic pain (CP) and cognitive decline (CD) are costly, challenging to treat, prevalent among older adults, and worsen each other over time. We are iteratively developing Active Brains-Fitbit (AB-F), a live video program for older adults with CP and CD that teaches mind-body skills and gradual increases in step count. AB-F has demonstrated feasibility; acceptability; and signs of improvement in emotional, physical, and cognitive functions when delivered in person to older adults.

**Objective:**

We are conducting a feasibility randomized controlled trial (RCT) of AB-F versus a time- and dose-matched educational control (health enhancement program [HEP]) in older adults with CP and CD. Here, we describe virtual adaptions to our study protocol, manualized treatments, evaluation plan, and study design in response to feedback from former participants and COVID-19. We will evaluate the feasibility benchmarks and the potential of AB-F to improve physical, emotional, and cognitive functions.

**Methods:**

This is a single-blind pilot RCT. Participants are randomized to AB-F or HEP. Patients are recruited through pain clinic referrals, institutional registries, and flyers. Interested participants are screened for eligibility via telephone and provide electronic informed consent. After randomization, participants are mailed all study documents, including their treatment manual, an ActiGraph accelerometer, and a Fitbit (separate envelope for AB-F only). Both conditions are manualized and delivered over 8 weekly sessions via Zoom. Participants complete self-report and performance-based (6-min walk test and Montreal Cognitive Assessment) outcome measures via Zoom at baseline and post intervention. Primary outcomes are a priori set feasibility (recruitment, quantitative measures, and adherence), acceptability, credibility, expectancy, and satisfaction benchmarks. Secondary outcomes are physical, cognitive, and emotional functions as well as intervention targets (social function, pain intensity, pain-specific coping, and mindfulness).

**Results:**

The trial is ongoing. We have recruited 21 participants (10 AB-F and 11 HEP) across 2 rounds. Only 2 participants have withdrawn (1 before baseline and 1 before the first session). All 19 remaining participants have completed the baseline assessment. In the first round, attendance is high (11 out of 12 participants completed all 4 sessions so far), and AB-F participants are adherent to their Fitbit and step goals (5 out of 6 participants).

**Conclusions:**

Preliminary findings are promising for the feasibility of our completely virtual AB-F intervention. However, these findings need to be confirmed at the trial conclusion. This study will answer important questions about the feasibility of delivering a completely virtual mind-body activity program to older adults with comorbid CP and CD, which, to our knowledge, is unprecedented. Details on integrating multiple digital platforms for virtual assessments and intervention delivery will inform treatment development for older adults and those with comorbid CP and CD, which is crucial during the COVID-19 pandemic.

**Trial Registration:**

ClinicalTrials.gov NCT04044183; https://clinicaltrials.gov/ct2/show/NCT04044183

**International Registered Report Identifier (IRRID):**

DERR1-10.2196/25351

## Introduction

### Background

Chronic pain (CP), or pain that persists for more than 3 months, is common in the United States, costly to the health care system, and difficult to treat [1]. CP becomes more prevalent with increasing age, affecting 25-50% of community-dwelling older adults [2] and over 80% of nursing home residents [3]. Cognitive decline (CD) [4], defined as subjective (ie, self-report only) or objective (ie, confirmed by formal testing) decreases in cognitive performance that surpass normal aging [2], is a growing public health priority as life expectancy increases. There is a bidirectional relationship between CP and CD [5]. Older adults with CP are twice as likely to endorse CD [4] and are at greater risk for neurodegeneration [6], which in turn exacerbates perceptions of CP [7]. CP [8,9] and CD [10] exacerbate each other, placing individuals on a *disability spiral* of worsened physical, emotional, and cognitive functioning [11,12].

Unfortunately, current treatments are inadequate for addressing the CP-CD comorbidity among older adults [4,13]. CP and CD are often initially treated with medications, which are limited in efficacy [14] (eg, lack cognitive benefits) [15]; increase the risk of adverse events, such as falls [16]; and are associated with harmful side effects that can worsen CD [17]. Nonpharmacological interventions for CP that teach adaptive coping skills can improve physical, emotional, and social functioning [18] but overlook the needs of older adults with CD. Walking-based mind-body activity programs may be feasible and effective in addressing the CP-CD comorbidity among older adults [19-22].

**Figure 1:**
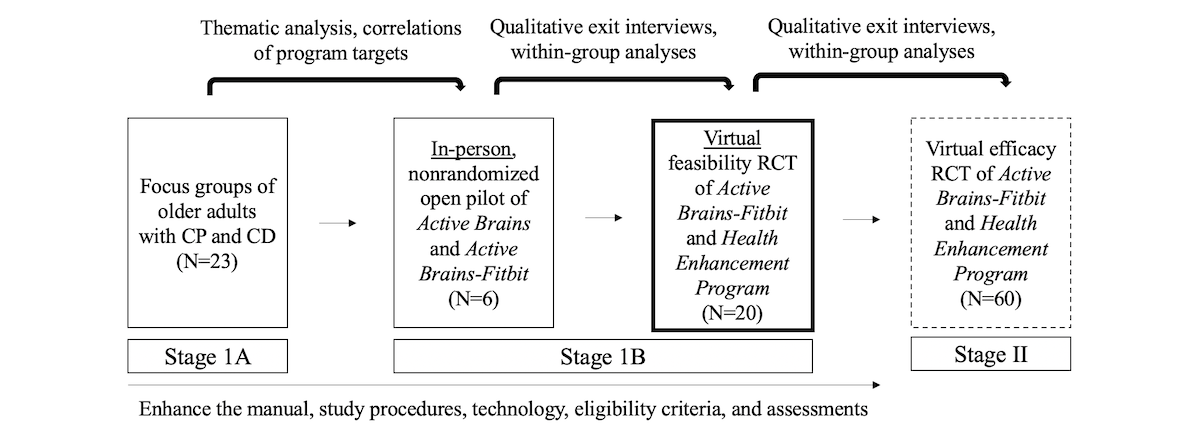
Iterative stages of Active Brains-Fitbit development. The study described in this protocol is outlined in bold. The subsequent efficacy randomized controlled trial is outlined by a dashed line. CD: cognitive decline; CP: chronic pain; RCT: randomized controlled trial.

We are iteratively developing the first mind-body activity program to address the CP-CD comorbidity among older adults using the National Institute on Aging (NIA) Stage Model [23], which emphasizes early refinement before efficacy testing (Figure 1). First, we developed Active Brains (AB) and Active Brains-Fitbit (AB-F) using qualitative data from patient focus groups (stage 1A) [13]. Both programs teach identical mind-body skills to address the CP-CD comorbidity and increase participants’ step count, but AB-F participants set individualized quota-based step goals [21] reinforced by a Fitbit [24]. In a nonrandomized open pilot trial (stage 1B), both programs similarly displayed (1) preliminary feasibility and acceptability when delivered in person; (2) within-group improvements for pain intensity, pain-specific coping, physical function, and cognitive function; and (3) high participant satisfaction [13]. Qualitative individual exit interviews assisted in further optimizing the program components and study methodology [13]. Participants in the Fitbit group found the device useful for monitoring their progress in real time, enhancing motivation, and reinforcing individualized goals, which align with positive perspectives of technology to modify health behaviors [25,26].

These findings informed 2 main decisions in the preparation for a future stage II efficacy trial. First, because AB and AB-F performed similarly with regard to both feasibility benchmarks and preliminary effects and participants in the AB-F group found using a Fitbit to monitor and safely increase step count beneficial, our next stage 1B trial will be a single-blind pilot randomized controlled trial (RCT) of AB-F versus an attention placebo control (health enhancement program [HEP]) [27]. Second, due to COVID-19, many of the exit interviews after our in-person trial were conducted virtually [28], and participants generally preferred this remote modality. Qualitative results from our previous work [29] highlighted participants’ interest in live video delivery to overcome barriers to in-person attendance commonly experienced by older patients, such as lack of flexible scheduling, difficulty coordinating transportation, and travel costs [30]. Further, a growing body of research shows that older adults can effectively use technology [31], including live video [32] and wearable devices. Our virtual adaptations to mind-body interventions for patients with neurofibromatosis [33,34], stroke, and CP [35] suggest that older adults with CP and CD [13] may also be amenable to AB-F delivered via live video. The findings will inform a subsequent pilot RCT to test feasibility benchmarks of the ability to randomize individuals to the intervention (AB-F) or control (HEP) as well as deliver the programs and conduct all study procedures virtually.

### Objectives

Here, we describe live video adaptations to study procedures and delivery of AB-F versus HEP in older adults with CP and CD within a single-blind RCT. We hypothesize that AB-F delivered via live video would meet a priori feasibility (recruitment, quantitative measures, and adherence), acceptability, credibility, expectancy, and satisfaction benchmarks similar to our in-person trial. Patients’ in-depth perceptions of technology will be assessed, including virtual assessment and intervention delivery via exit interview focus groups with participants after the programs as well as through a post intervention self-report survey. The results will inform a subsequent efficacy RCT (stage II) of AB-F versus HEP, both delivered in group via live video. The following hypotheses will be tested: (1) AB-F is superior to HEP in improving objective, performance-based, and self-reported measures of physical, cognitive, and emotional function outcomes; (2) AB-F–related improvements will sustain over time; and (3) program targets (eg, mindfulness and coping) and relevant clinical and demographic variables will serve as mediators and moderators of improvement in outcomes.

## Methods

### Study Design and Setting

**Figure 2:**
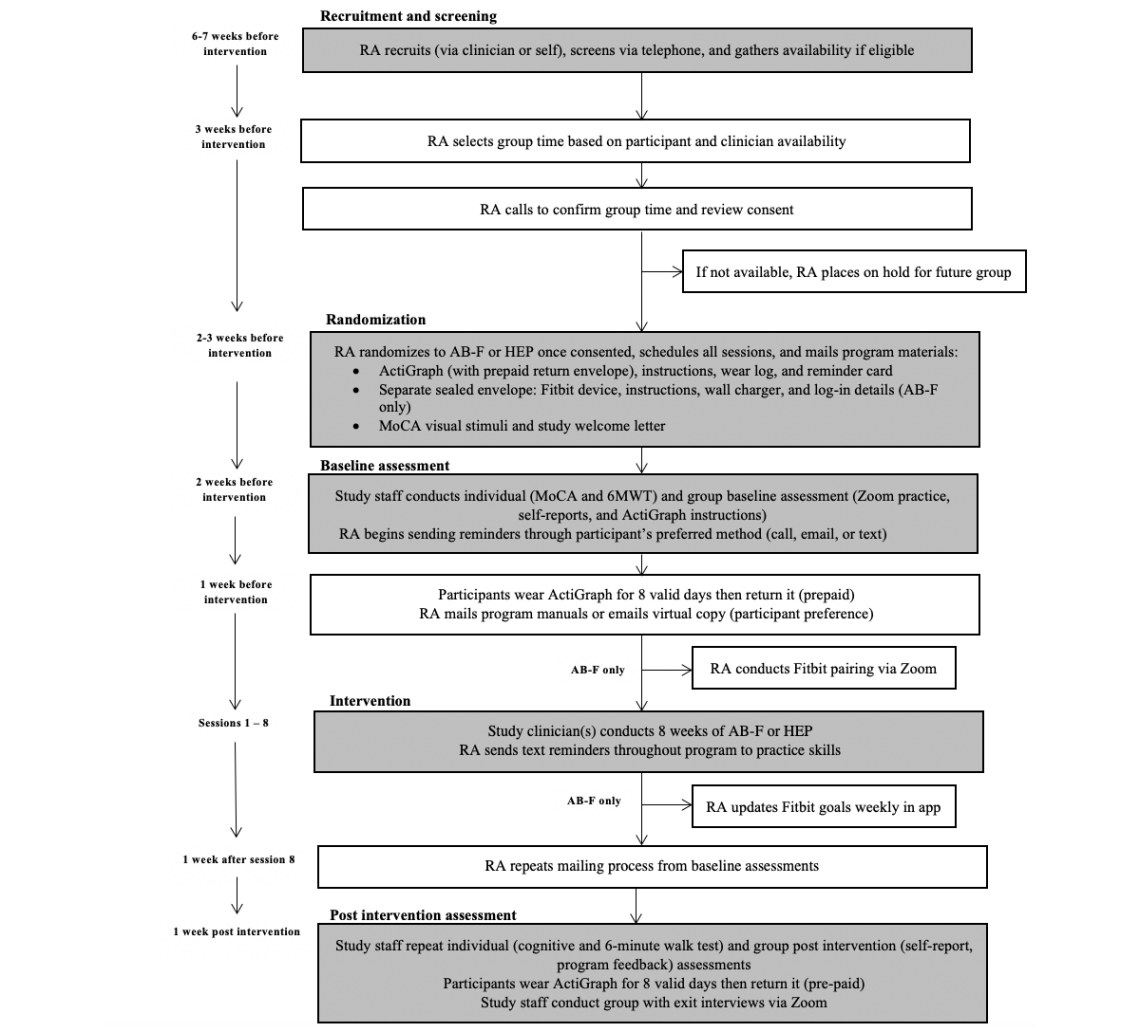
Study design and timeline. AB-F: Active Brains-Fitbit; HEP: health enhancement program; MoCA: Montreal Cognitive Assessment; RA: research assistant; and 6MWT: 6-min walk test.

This single-blind feasibility RCT of AB-F versus a time- and dose-matched educational control (HEP) in older adults with heterogeneous CP and CD is being conducted at a large academic medical center in the Northeastern United States. Our institutional review board (IRB) approved this study (#2018P002152). Figure 2 presents a diagram of the study design and timeline of the procedures outlined below.

### Inclusion and Exclusion Criteria

Textboxes 1 and 2 present the criteria and rationale for study inclusion and exclusion, respectively. These eligibility criteria are consistent with similar mind-body trials with patients with CP [18,35] and our earlier program development work with this population [13,29]. The criteria are meant to be as inclusive as possible by allowing individuals with any type of musculoskeletal CP and any type of subjective or objective CD to participate to maximize generalizability consistent with NIA guidelines [23].

**Study inclusion criteria and rationale.**

Inclusion criteria and rationale

- Male and female outpatients, aged 60 years or older. Population under study
- Have nonmalignant chronic pain for more than 3 months. International Association for Study of Pain [36] criteria
- Self-report cognitive decline, such as forgetting names or obligations, getting lost, and having to repeat information. Population of study
- Able to perform a 6-min walk test at an accelerated pace. Program will involve increasing the number of steps for the primary physical function outcome measure
- Free of concurrent psychotropic or pain medication for at least 2 weeks before initiation of treatment or stable on current psychotropic or pain medication for a minimum of 6 weeks and willing to maintain a stable dose. Treatment confound
- Cleared by a medical doctor for study participation and no self-reported concerns about physical functioning on the Physical Activity Readiness Questionnaire [37]. Human subject concern, risk
- Has access to a smartphone with Bluetooth 4.0 capability to enable the Fitbit device and 6-min walk test (*Timed Walk*) [38] app and a computer for video software (Zoom for remote assessments and treatment sessions). Necessary for pairing with Fitbit and storing/downloading data, conducting physical function assessments remotely, and virtual group sessions

**Study exclusion criteria and rationale.**

Exclusion criteria and rationale

- Diagnosed with a medical illness expected to worsen in the next 6 months (eg, malignancy). Treatment confound
- Serious mental illness or instability for which hospitalization may be likely in the next 6 months. Feasibility, participant safety
- Self-reported current suicidal ideation. Participant safety
- Lifetime history of schizophrenia, bipolar disorder, or other psychotic disorder. Treatment confound
- Current substance abuse or dependence and current substance use disorder, within the past 6 months. Treatment confound
- Practice of yoga/meditation, or other mind-body techniques, once per week for 45 min or more within the last 3 months or less. Treatment confound
- Regular use of Fitbit in the last 3 months. Treatment confound
- Engage in regular intensive physical exercise for more than 30 min a day. Treatment confound
- Unable to walk without the use of assistance (eg, walker, cane, and wheelchair). Treatment confound

### Recruitment and Screening

Participant recruitment and screening was initiated in August 2020. To facilitate local recruitment of older adults with comorbid CP and CD, we established interdisciplinary partnerships with the Memory Disorders Division, Center for Pain Medicine, the Psychological Assessment Center, and the Osher Center for Integrative Medicine Clinical Program within our institution. Participants may also present to hospital-affiliated or regional medical practices that treat CP or CD and meet the study criteria. Our IRB-approved recruitment flyer is distributed to physicians at these recruitment sites and public online groups for CD and/or CP (eg, open forums for CD and Facebook groups for individuals with CD and their loved ones). Use of virtual recruitment and enrollment as well as live video intervention delivery allows geographically diverse older adults to participate in the study.

A trained research assistant with experience in coordinating mind-body intervention trials for CP recruits and screens participants by phone from a private location. The research assistant provides study details to interested participants and screens for eligibility via phone. Those who express interest and wish to participate in the study may opt to review the consent form briefly with a member of the study staff via phone during the initial screening conversation. The research assistant makes 3 contact attempts before discontinuing and maintains an updated log of all screening attempts for study data reports. The principal investigator, a licensed clinical health psychologist with expertise in older adults, mind-body interventions, CP, and CD, reviews all cases before enrollment to confirm eligibility. We have successfully used this strategy in prior intervention development trials conducted remotely [39,40].

### Enrollment

Our goal was to enroll and randomize up to 10 participants for each of the 2 rounds in this pilot RCT (N=20) and to deliver the programs in small groups of 5-6 participants, consistent with guidelines for conducting virtual group interventions [41,42]. The research assistant coordinates with eligible and interested participants via phone to select an appropriate time for group meetings based on the availability of the majority of participants. The research assistant emails participants the consent form and asks them to return an electronically signed copy within 48 hours. If needed, the research assistant contacts participants to answer remaining questions about the consent form (ie, how to electronically sign). Participants are considered enrolled when they have returned the signed informed consent form via email, are randomized, and attend at least one session. Participants earn US $30 for each assessment (baseline and post intervention, US $60 in total), US $10 for each intervention session (8 sessions, US $80 in total) and homework (AB-F only), and US $30 for the exit focus group (US $170 in total).

### Randomization to Treatment Arm

Randomization occurs after consent but before the baseline assessment to allow time for mailing the Fitbit to those in AB-F. Randomization follows a block design (blocks of 12) to ensure that equal numbers of patients are split into the AB-F or HEP groups. To maintain single-arm blinding, the study staff refer to the AB-F and HEP as AB1 and AB2, respectively. After randomization, the research assistant sends the Zoom appointment information for group sessions for the 10 weeks of the study, including the following: (1) the baseline assessment to practice Zoom, receive accelerometer instructions, and complete self-reports via Research Electronic Data Capture (REDCap); (2) 8 intervention sessions; and (3) post intervention to readminister self-reports and review accelerometer instructions. The research assistant also mails each participant a package that contains a folder with: (1) a welcome letter from the principal investigator (AV), (2) testing materials for the Montreal Cognitive Assessment (MoCA) [43] and accelerometer (wear-time log, instructional document, and reminder card), and (3) a prepaid envelope to mail the accelerometer back to the study staff. The AB-F group receives an additional sealed envelope with a Fitbit, charger, wall-plug, instructions on the device, and log-in information. AB-F participants are asked to not open that envelop until their Fitbit pairing session, and all participants are notified of their group assignment after all baseline assessments are complete.

### Live Video and Technology Considerations for Older Adults With CP and CD

Prior research shows that older adults face several barriers to adopting new technology, including decreased learning and memory capacity, lower self-efficacy, and decline in vision and motor skills [44-47]. To optimize feasibility, acceptability, and adherence, we follow guidelines for facilitating older adults’ use of technology, such as leveraging social support [48], providing reassurance, and linking to personal relevance [49,50], and allowing time for self-directed learning and experimentation to develop confidence [51]. We use additional strategies to further promote familiarity with the specific technologies used in this virtual RCT. First, the research assistant gauges individualized levels of technical support needed by asking participants: (1) whether they have used Zoom before, (2) which laptop and smartphone devices they own, (3) if they have an in-person support who can help them troubleshoot, and (4) whether they prefer an online or physical copy of the program manual. Second, we instruct participants to contact study staff for technological assistance at any time. Third, the study clinician and research assistant collaborate via text messaging to provide real-time technical support during session appointments (eg, connection or audio/video issues). Fourth, the research assistant immediately contacts participants who missed a group session to schedule a make-up with the study clinician to prevent missed material. Fifth, participants in both groups may consent to electronic reminders (phone calls, text messages, or email based on preference) to increase session attendance and adherence to technology. Text messages are sent once a day during the study, and participants may opt out at any point. Sixth, the study clinician allots up to 10 min at the start of each session to overcome technological barriers that emerge. We describe specific live video adaptations to our procedures using the technologies below.

#### Live Video Delivery

We use live video (Zoom) for all study procedures, including assessments and intervention delivery. We developed the live video procedures using our experience in delivering virtual mind-body programs in prior studies [39,52,53] and consultations with the Society of Behavioral Medicine Behavioral Informatics Special Interest Group. The research assistant sends download instructions to participants who are unfamiliar with Zoom and offers individualized technical support as needed. Two weeks before the first treatment session, the research assistant schedules a 90-min group baseline assessment via Zoom with all participants and study staff to provide a tutorial and explain the accelerometer and self-report baseline assessments (refer to the Assessment Procedures below). During this baseline assessment, the study staff guide participants in enabling their audio/video and positioning their camera appropriately. In case multiple participants encounter technical challenges at once, additional research assistants are on standby for the duration of the call. Participants learn the procedures and rationale for using the following Zoom functions during group sessions: gallery view to see all participants, camera mode to enable video, mute to limit noises in their environments when not speaking, and host mute capabilities in the event that participants cannot mute themselves or forget to do so when appropriate. Participants are also informed of the privacy features of Zoom (eg, encryption and password protection) and that sessions will be audio recorded.

#### Fitbit Step Count

Participants in the AB-F group receive their Fitbit, charger, wall-plug, Fitbit account information, and user manual via mail in a separate sealed envelope. Following the 1-week baseline accelerometer assessments, all participants in the AB-F group meet the research assistant via Zoom to pair their Fitbit to a Bluetooth-enabled smartphone. Participants are instructed to keep their device in a safe location or charging until the first group session. Participants wear the Fitbit from the first session to post intervention (except while bathing). Fitabase, a secure web-based data collection platform, allows the research assistant to remotely monitor participants’ daily Fitbit data for adherence and to ensure that the Fitbit is not being worn before the first session (to prevent biasing the baseline assessment). The research assistant sets AB-F participants’ weekly walking goals, which appear on their watch and smartphone app, by logging into their Fitbit account on a computer. Participants are sent weekly emails with their updated walking goal, based on the goal set the previous week and whether or not the goal was met. The research assistant also sends weekly text message reminders to charge and synchronize the device.

#### Accelerometer Step Count

After consenting and randomization, participants receive a wGT3X-BT ActiGraph accelerometer [54] in the mail and a folder that contains a wear log, a reminder card, and simple instructions with photos to properly wear the device. During the group baseline, the study staff asks participants to open the mailed envelope to review the accelerometer procedures and discuss solutions to common issues (eg, forgetting to wear the device and interference with clothing) detailed in our prior qualitative work [13]. All participants wear the accelerometer over their right hip, log each time they put the device on and take it off, and record their daily walking or any other exercise (to corroborate the objective step count data) for 8 days. Participants then return the accelerometer using a prepaid envelope. The research assistant provides daily reminders to wear the accelerometer and complete the log using the participants’ preferred method of contact (phone, email, or text). The research assistant uses these check-ins, along with data monitoring in the ActiLife software [55], to confirm that all participants record 8 days (ie, 1 week plus the day of the assessment) of valid accelerometer wear (≥7 hours/day) at baseline and post intervention. Participants with 5 or fewer valid days are either given an extension before returning the accelerometer back or are mailed the device again. Our mailing procedure is similar to the recent accelerometer protocols [56].

#### 6-Min Walk Test

Participants complete the 6-Min Walk Test (6MWT) [57] using an app (*Timed Walk*) on their smartphone [38] at baseline and post intervention. *Timed Walk*, which measures walking distance within a fixed timeframe using smartphone-based GPS, is a valid performance-based measure of physical function and is a reliable alternative to traditional laboratory assessments [38]. Study staff assist participants with downloading *Timed Walk* via the app store during an individual Zoom session (15 min). Participants are instructed to self-administer the 6MWT using *Timed Walk* by walking outside on flat terrain and emailing or calling the research assistant to submit their results. To ensure safety and increase adherence, participants create a plan to complete the 6MWT on a familiar route at a specific time and date with support from a friend or family member for safety (eg, driving the participant to a familiar area) or technology (eg, navigating the app and submitting the results), if needed. We protect participants’ privacy by providing information about the GPS location and steps data collected during consent and recommend deleting the app until the post intervention assessment.

#### MoCA

Study staff also administer the MoCA [43] following audio-visual guidelines [58] at baseline and post intervention during the individual Zoom session (before completing the 6MWT). We instruct participants via email to have the visual stimuli (mailed in the packet of study materials) and a pen ready for the virtual MoCA administration. Participants hold their answers to the first 3 MoCA items (trails, cube, and clock) to the video camera for study staff to screenshot and score.

#### Self-Report Measures

Participants complete questionnaires online via REDCap [59] during the group assessments (baseline and post intervention) via Zoom. The research assistant emails participants a secure link to complete the questionnaires. The research assistant aids participants in accessing their email and clicking on the REDCap link while remaining connected to Zoom. The study clinician mutes all participants to aid focus during the completion of the questionnaires. Participants are encouraged to use the *hand raise* function on Zoom or temporarily unmute themselves for technical support or to ask clarifying questions about the self-reports as needed. The research assistant remains on the Zoom call and monitors participants’ questionnaire completion status using the REDCap dashboard but does not influence their responses. If participants encounter significant difficulties that prevent them from completing the questionnaires during the baseline session (eg, due to technology or CD symptoms), the research assistant schedules a call with the participant the following day to troubleshoot and ensure that all questionnaires are completed. Study staff review all questionnaires for missing data and errors that were not prevented by the REDCap response validation features.

### Treatment Arms

The 8 treatment and 2 assessment sessions (all 90 min) are delivered to both treatment arms concurrently via Zoom over a total of 10 weeks. Participants can attend the online group sessions from their home or another private place with a personal computer. Both treatment arms are delivered by trained clinicians under the direct supervision of health psychologists with expertise in mind-body and walking interventions, geropsychology, and CP. We follow the National Institutes of Health recommendations [60] and our previously developed clinical adherence protocol to assess treatment fidelity of both programs [39]. The clinicians complete fidelity checklists after each session and undergo weekly supervision to reinforce protocol adherence. We will confirm fidelity to both programs by independently coding adherence in a random sample (10%) of the audio recorded sessions. Table 1 outlines the 8 AB-F and HEP sessions.

**Table 1:** Session outline for the Active Brains-Fitbit and health enhancement program for older adults with chronic pain and cognitive decline.

Session	AB-F^a^ topic	AB-F skills and session content	HEP^b^ topic	HEP skills and session content
1	CP^c^ and CD^d^: the disability spiral	Myths about pain, unhelpful pain alarm, disability spiral, mind-body connection, deep breathing, and gratitude practice	Program overview and CP and CD	Understanding CP and CD, connection between CP and CD, and impact of stress
2	“Walk All Over” the disability spiral	Quota-based walking, choosing meaningful activities, setting a walking plan, education on increasing daily walking, self-compassion, and barriers to using the Fitbit	The connection between CP, CD, and physical wellness	Connection between CP and CD
3	Mindfulness and pain	Mindfulness, breathing meditation, body scan, mindful moments, and pain awareness	Sleep and wellness	Sleep hygiene, cognitive and physical health
4	Mindfulness of pain sensations	Noticing unhelpful alarms, mindfulness of pain meditation, mindful walking, and overcoming barriers to walking	Exercise and wellness	Physical exercise, maintaining healthy weight, and tips for getting active
5	Building cognitive reserve	Education on cognitive abilities, CP-CD connection, coping with cognitive difficulties, engaging your intellect, and cognitive mindful moments	Nutrition I: the basics	Basic nutrition, portion size and calories, and understanding food labels
6	Strengthening social support for CP and CD	Social support and the pain cycle, types of social support, reducing loneliness: get active together, social support in CP and CD, and effective communication	Nutrition 2: healthy weight and weight loss	Eating healthier meals and snacks, eating out healthy, and weight loss and BMI
7	Coping skills to get back on track	CP, CD, and values; why we walk; getting back on track with walking; and stop and breathe, reflect, and choose	Managing your health care for CP and memory-related problems	Communicating with doctors, health diary, medical emergencies, and medication adherence
8	Staying on track and maintaining your progress	The powerful self, staying on track, reflecting on Active Brains skills, and resiliency plan	Review of Active Brains 2	Overview of program skills

^a^AB-F: Active Brains-Fitbit.

^b^HEP: health enhancement program.

^c^CP: chronic pain.

^d^CD: cognitive decline.

#### AB-F

Full details on the AB-F program can be found in our prior work [13]. Briefly, AB-F encourages gradual increases in daily step count through individualized goal setting using quota-based (eg, meeting a step goal of 5000 steps) rather than pain-contingent walking, reinforced by Fitbit [24]. AB-F also targets the CP and CD comorbidity by teaching mind-body; pain-cognition awareness; as well as cognitive, emotional, and social functioning skills. On the basis of the qualitative results from our stage 1B exit interviews, we enhanced the AB-F manual to (1) increase education and time spent on mindfulness, gratitude, and self-compassion skills; (2) strengthen goal setting through simplified walking plans that prioritize repetition and problem solving; (3) improve brain health education and practical strategies for compensating with CD; (4) enhance sensitivity to visual impairments by streamlining the text, adding visuals, and using an age-friendly font (type and size); and (5) refine the final session on maintaining progress beyond the program. We further modified specific program components directly impacted by COVID-19, including walking (eg, emphasizing outdoors and physically distant locations and wearing a mask), cognitive (eg, maintaining mental stimulation during quarantine), and social skills (eg, using technology to remain connected and reducing loneliness). Participants email their homework log that documents their walking progress, mind-body and gratitude practice, and pain ratings by the morning of each session for the study clinician’s review (AB-F only).

#### HEP

Our previous work provides full details on HEP [27]. Briefly, this active control accounts for the effect of time spent as well as feedback and support from group members and the study clinician. Participants receive lifestyle education consistent with public health recommendations and standards for health promotion (eg, physical activity, sleep, nutrition, healthy weight, and medical appointments). The program has been successfully used as an active control in multiple prior studies [33,39,40]. We adapted the HEP to include population-specific information on CP and CD symptoms. Reminders to practice the mind-body and activity skills in AB-F are matched with reminders of the health education learned for the HEP.

### Feasibility Markers

Table 2 contains the a priori set benchmarks and criteria that align with our prior in-person trial [29] and similar feasibility pilot studies [35,39,61]. We will assess the feasibility (recruitment, quantitative measures, and adherence), acceptability, credibility, expectancy, and satisfaction of both programs delivered virtually.

**Table 2:** Feasibility and acceptability of the programs.^a^

Marker	Description	Criteria
Feasibility of recruitment	Proportion of patients who agreed to participate from the total contacted	Excellent: ≥80% of contacted patients agree to participate Good: ≥70% of contacted patients agree to participate
Program acceptability	Proportion of participants who attended 6 out of 8 sessions (including makeups)	Excellent: ≥80% of participants attend 6 out of 8 sessions Good: ≥70% of participants attend 6 out of 8 sessions
Credibility and expectancy	Proportion of participants above the Credibility and Expectancy Questionnaire [62] midpoint	Excellent: ≥80% of participants rate credibility and expectancy above the scale midpoint Good: ≥70% of participants rate credibility and expectancy above the scale midpoint
Therapist adherence to manual	Clinician adherence to audio recording, progress note, and checklist with content delivered	Excellent: 100% of audio recordings, progress notes, and checklists were completed with 100% of content delivered Good: ≥75% of audio recordings, progress notes, and checklists were completed with 100% of content delivered
Feasibility of quantitative measures	Number of questionnaires entirely missing in more than 25% of participants	Acceptable: No questionnaires were entirely missing in >25% of participants and or had an internal reliability below 0.70
Adherence to homework	Proportion of participants who completed at least 5 out of the 7 homework logs	Excellent: ≥80% of participants complete at least 5 out of the 7 homework logs Good: ≥70% of participants complete at least 5 out of the 7 homework logs
Adherence to ActiGraphs and Fitbit	Number of participants with valid ActiGraph data (≥7 hours) for 6 out of 8 days; number of participants who wore the Fitbit for 5 out of 7 days. We also report the number of days participants step count goal was met	Excellent: ≥80% of participants with valid ActiGraph data on ≥6 out of 8 days per week Good: ≥70% of participants with valid ActiGraph data on ≥6 out of 8 days per week Excellent: ≥80% of participants wear the Fitbit at least 5 of the 7 days per week Good: ≥70% of participants wear the Fitbit at least 5 of the 7 days per week
Modified patient global impression of change	Participant ratings of overall improvement in program outcomes	Lower scores reflect higher amounts of perceived improvements
Client satisfaction	Proportion of participants above the Client Satisfaction Questionnaire midpoint [63]	Excellent: ≥80% of participants rate satisfaction above the scale midpoint Good: ≥70% of participants rate satisfaction above the scale midpoint
Program safety and adverse events	Number of adverse events reported by participants throughout the program	Excellent: no adverse events linked to program participation are reported and considered Good: mild adverse events are reported in ≤10% of participants linked to program participation

^a^We set benchmarks based on development guidelines [64,65] and our feasibility pilots [29,35,39,61].

### Quantitative Assessments

We selected quantitative measures informed by the CP and CD literature and by our prior mixed methods study [13,29], which provided preliminary evidence for signals of improvement in this population. Following the Initiative on Methods, Measurement, and Pain Assessment in Clinical Trials criteria [66], we measured physical function comprehensively with an objective measure (accelerometer step count), a performance-based measure (6MWT using *Timed Walk*), and several self-report measures (questionnaires). Textbox 3 provides brief descriptions of all quantitative assessments.

**Study measures and constructs.**

Demographics

- Date of birth, gender, weight, height, handedness, race/ethnicity, marital status, educational level, employment status, occupation, income, pain diagnoses, length of chronic pain, comorbid medical conditions, current/history of mental health condition, current pain medication, and brain health lifestyle behaviors. *Pre*

Pain

- Numerical Rating Scale; measures pain intensity at rest and during activity. *Pre and Post* [67]
- Use of rescue analgesics. *Weekly homework log and self-report*

Physical function: self-reported

- World Health Organization Disability Assessment Schedule 2.0: measures for disability in 6 domains: cognition, communication, transportation, self-care, daily responsibilities, and engaging in community activities. *Pre and Post* [68]
- Patient-Reported Outcomes Measurement Information System (PROMIS) Physical Function v.1.2.8b; assesses level of difficulty with daily function. *Pre and Post* [69]
- The Godin Leisure-Time Exercise Questionnaire: measures the number of times per week physical activity with different intensities (light, moderate, and strenuous) is performed. *Pre and Post* [70]

Physical function: ambulatory (objective)

- Accelerometer (ActiGraph) [54]: measures activity during 8 days in terms of number of steps. *Pre and Post*

Physical function: performance-based

- 6-min walk test via the *Timed Walk* app: assesses distance walked at a fast pace in 6 min in meters using smartphone GPS. *Pre and Post* [38]

Cognition: objective

- Montreal Cognitive Assessment: measures cognitive domains (ie, attention, concentration, executive functions, memory, language, visuospatial skills, abstraction, calculation, and orientation) used to detect level of cognitive decline. *Pre and Post* [43]

Cognition: self-reported

- Everyday Cognition Scale: assesses cognitive functioning by comparing with current performance on cognitive tasks to a decade ago. *Pre and Post* [71]

Emotional function

- PROMIS depression, v1.0.8b: assesses negative mood, views of self, engagement in daily living, and social components. *Pre and Post* [72]
- PROMIS anxiety, v1.08a: assesses fear, worry, hyperarousal, and somatic symptoms. *Pre and Post* [72]

Social functioning

- PROMIS emotional support v4a: assesses level of perception of having close relationships. *Pre and Post* [73]
- UCLA Loneliness Scale: assesses level of perception of isolation. *Pre and Post* [74]

Pain-specific coping

- Pain Catastrophizing Scale: assesses hopelessness, helplessness, and rumination about pain. *Pre and Post* [75]
- Pain Self-Efficacy Questionnaire: measures level of self-efficacy for performing activities of daily living despite pain. *Pre and Post* [76]

General coping

- Measures of Current Status: assesses ability to engage in a series of general healthy coping skills (eg, relaxation, being aware of tension, expressing needs, confidence in coping, and assertiveness). *Pre and Post* [77]
- Cognitive and Affective Mindfulness Scale-Revised: assesses usage of mindfulness skills. *Pre and Post* [78]
- Gratitude Questionnaire: measures ability to experience daily gratitude. *Pre and Post* [79]
- Self-Compassion Scale: measures level of how understanding individuals are able to be to themselves in a stressful situation. *Pre and Post* [80]
- Tampa Kinesiophobia Scale: measures extent of impact on physical activity due to fear of pain or injury. *Pre and Post* [81]
- Chronic Pain Acceptance Questionnaire: measures the level in which one is able to engage in activity, despite their pain. *Pre and Post* [82]

### Exit Focus Group Procedures

All participants will have the opportunity during the post intervention REDCap survey to provide feedback on the program via Likert questionnaires and open responses on the study technology (Zoom, virtual MoCA, and *Timed Walk*), Fitbit (AB-F group only), procedures, treatment manuals, support from study staff, and expectations of the program. In addition, we will conduct a brief virtual exit interview focus group (30 min) via Zoom during the post intervention assessments with both AB-F and HEP participants to further explore the impressions of the program and inform the next trial. Given prior optimization of the program via qualitative methods, the exit focus groups will gather impressions about the virtual delivery of skills and the technological aspects of the program. We will follow our procedures for conducting virtual focus groups [83] and guidelines for collecting qualitative data [84,85].

### Data Analysis

Consistent with guidelines for early feasibility studies [86,87] and the NIA Stage Model, our mixed method analysis will not assess efficacy [23]. However, we will evaluate whether this virtual pilot RCT achieved similar feasibility and acceptability to our prior in-person trial [29]. Our target sample size is appropriate for exploring feasibility and outcomes for future trials [86] and is consistent with our previously published pilot studies [29,35,39,61]. The frequency and proportions of the feasibility benchmarks will be calculated separately for AB-F and HEP. Additional quantitative analysis will focus on descriptive statistics for each measure, within-group pre-post comparisons using paired *t* tests, Cohen *d* effect sizes to explore signals of improvement in AB-F, and exploratory correlations between outcomes (physical, cognitive, and emotional function) and program targets (eg, mindfulness and coping). Qualitative analysis will be primarily deductive [88] using the framework method based on our prior work [13], allowing for some inductive flexibility to explore the unexpected needs and preferences of participants [89].

## Results

The trial is ongoing. As of October 2020, we have recruited 21 participants (10 AB-F and 11 HEP) across 2 rounds of groups. One participant dropped before the baseline assessment (scheduling conflict) and 1 dropped before the first AB-F session (technology barriers and receiving surgery). All 19 remaining participants have completed the baseline assessment. In the first round of groups, attendance is high (11 out of 12 participants completed all 4 sessions so far). AB-F participants are adherent to their Fitbit (5 out of 6 participants wore the device at least 6 out of 7 days all 4 weeks), and 5 out of 6 participants have met their weekly step goals for at least half (2) of the sessions conducted so far (4). We have retained 2 participants who underwent a medical procedure (1 shoulder surgery and 1 skin cancer surgery) unrelated to the program.

## Discussion

### Scientific Contribution

CP and CD are frequently comorbid among older adults [4]. CP symptoms exacerbate CD and vice versa [5], leading to a *disability spiral* of worsened physical, cognitive, and emotional functioning [11,12]. The AB-F program addresses an important clinical gap, as no effective treatments are currently available for this population. The 2 AB-F development studies conducted thus far provide preliminary evidence that combining mind-body and activity skills with Fitbit is feasible; acceptable [13,29]; and shows promise for improving physical, cognitive, and emotional outcomes among older adults with CP and CD. This protocol provides a blueprint for an entirely virtual, single-blind feasibility RCT of AB-F versus a time- and dose-matched educational control (HEP) in older adults with CP and CD. Importantly, our technological adaptations are consistent with older patients’ evolving preferences for live video delivery and bypass barriers to nonpharmacological treatments identified in the literature [30] and older adults in our prior studies (eg, transportation) [13,29] as well as the recent threat of COVID-19. To our knowledge, this is the first trial to integrate a live video, a smartphone, and wearable technologies to enhance treatment development for older adults with CP and CD.

This mixed methods study will help us maximize the feasibility, credibility, acceptability, and adherence of the AB-F and HEP programs. Quantitative and qualitative data will be integrated to corroborate the feasibility of AB-F, contextualize the findings at multiple levels (group and individual participant), explore whether technological adaptations helped or hindered participation, and understand changes in the outcomes [90]. The results will inform a subsequent virtual efficacy RCT (NIA stage II; Figure 1). In the future efficacy RCT, we will test our hypothesis that AB-F is superior to HEP in improving physical, cognitive, and emotional functioning in older adults with CP and CD. In the fully powered trial, we will test the mechanistic hypotheses that AB-F indirectly improves these outcomes through additional targets, such as increasing mindfulness, self-compassion, and pain resilience, while decreasing pain catastrophizing and kinesiophobia.

### Preliminary Findings

Although this trial is ongoing, preliminary findings are promising for the feasibility of both programs and our study methodology conducted virtually. Older adults with CP and CD appear to be able to engage in remote data collection and live video group participation, including the use of multiple technology platforms (ActiGraph, Fitbit, and Zoom). This suggests that our protocols for recruitment and teaching technology as well as our overall methodology show promise. Direct participant feedback will help us further address the technological challenges that our target population might experience. However, qualitative studies [91], including our prior work in this population [13,29], suggest that older adults are motivated to learn the live video [32] and wearable [92,93] technology used in this study. Our exit focus groups and *lessons learned* from study staff will help us develop further strategies to make the multiple technologies used during the program more accessible to older adults with CP and CD.

### Limitations

Despite the novelty of our entirely virtual mind-body and activity program, there are several limitations. First, our recruitment was restricted by the racial and ethnic distribution of patients at our pain clinic and memory clinic. Our future efficacy RCT will need to focus specifically on recruiting a sample that is representative of the US racial and ethnic composition by ensuring that we approach all racial and ethnic minorities or engage in targeted recruitment at the national level. Second, we did not formally assess the level of cognitive impairment at screening. Although no participants had a baseline MoCA score indicative of dementia (<18) [94], we plan to administer the Portable Mental Health Questionnaire [95] in future trials to screen for severe CD that would interfere with the programs or study procedures.

### Conclusions

Consistent with the early stages of the NIA model [23], optimizing our remote delivery procedures before conducting the virtual efficacy RCT is critical for ensuring feasibility, aligning the AB-F with our target population, and detecting meaningful changes [87]. If successful, the AB-F will be the first completely virtual intervention for older adults with CP and CD and can be routinely incorporated into telehealth practices. The need for nonpharmacological interventions that are amenable to remote delivery, such as mind-body and activity programs, has grown in response to COVID-19. We hope that in-depth descriptions of live video adaptations of study procedures will assist researchers conducting virtual clinical trials of similar programs for in-need populations.

## Appendix

Multimedia Appendix 1 Peer review report by the National Center for Complementary and Integrative Health.

## Abbreviations

AB: Active Brains
AB-F: Active Brains-Fitbit
CD: cognitive decline
CP: chronic pain
HEP: health enhancement program
IRB: institutional review board
MoCA: Montreal Cognitive Assessment
NIA: National Institute of Aging
PROMIS: Patient-Reported Outcomes Measurement Information System
RCT: randomized controlled trial
REDCap: Research Electronic Data Capture
6MWT: 6-min walk test
