# Supplementary material for: Live Video Adaptations to a Mind-Body Activity Program for Chronic Pain and Cognitive Decline: Protocol for the Virtual Active Brains Study
Source: JMIR Res Protoc. 2021 Jan 4;10(1):e25351. doi: 10.2196/25351 (PMC7813630; doi:10.2196/25351)
Supplement: Multimedia Appendix 1 [file resprot_v10i1e25351_app1.pdf]

SUMMARY STATEMENT

(Privileged Communication)

PROGRAM CONTACT:
Dr. Lanay Mudd
301-594-9346
muddlm@mail.nih.gov

Release Date: 03/06/2017
Revised Date:

Application Number: 1 R34 AT009356-01A1

Principal Investigator

VRANCEANU, ANA-MARIA

Applicant Organization: MASSACHUSETTS GENERAL HOSPITAL

Review Group: ZAT1 VS (02)
National Center for Complementary and Integrative Health Special Emphasis Panel
Exploratory Clinical Trials of Mind and Body Interventions Study Section

Meeting Date: 02/16/2017
Council: MAY 2017
Requested Start: 07/01/2017
RFA/PA: PAR14-182
PCC: MUDDL

Project Title: Integrating Mind Body Skills with Physical Activity to Improve Pain Outcomes in Patients with Heterogeneous Chronic Pain Conditions
SRG Action: Impact Score:28
Next Steps: Visit http://grants.nih.gov/grants/next_steps.htm
Human Subjects: 30-Human subjects involved - Certified, no SRG concerns
Animal Subjects: 10-No live vertebrate animals involved for competing appl.
Gender: 1A-Both genders, scientifically acceptable
Minority: 1A-Minorities and non-minorities, scientifically acceptable
Children: 3A-No children included, scientifically acceptable
Clinical Research - not NIH-defined Phase III Trial

Project Year	Direct Costs Requested	Estimated Total Cost
1	150,000	256,500
2	175,000	299,250
3	125,000	213,750
TOTAL	450,000	769,500

ADMINISTRATIVE BUDGET NOTE: The budget shown is the requested budget and has not been adjusted to reflect any recommendations made by reviewers. If an award is planned, the costs will be calculated by Institute grants management staff based on the recommendations outlined below in the COMMITTEE BUDGET RECOMMENDATIONS section.

1R34AT009356-01A1 VRANCEANU, ANA-MARIA

RESUME AND SUMMARY OF DISCUSSION: The applicant in this resubmission propose to examine and compare feasibility of a multimodal mind-body skills program adapted for patients with chronic pain (relaxation response resiliency program, p3RP) with a mind-body skills program that is integrated with use of a Fitbit digital monitoring device (DMD) and quota-based walking program (p3RP-DMD). The proposed project addresses the very prevalent and costly problem of chronic pain, which has become a national priority. The innovation resides in combining mind body programs with objective physical activity monitoring devices to improve physical and emotional variables related to chronic pain. The experimental design is well-written and based on a clear conceptual model, with well-defined specific aims. The Approach is well-thought out and methodologically rigorous, with appropriate choice of quantitative measures. The strengths of this application include the focus on chronic musculoskeletal pain, a problem of high clinical significance; the overall concept of integrating physical activity as a treatment target and integrating the Fitbit; the feasibility of the proposed research design; clear links with future work, providing an important and thoughtful platform on which to scale a larger trial; intervention and choice of outcomes aligned with NCCIH priorities and current recommendations in pain field (IMMPACT group); well-articulated methods for assessing feasibility including key milestones; appropriate quantitative measures and rigorous qualitative data collection and analysis; the highly qualified investigative team with the experience and expertise in pain, mind-body interventions, and clinical research. The research environment is outstanding and well-connected to Massachusetts General Hospital and affiliated Harvard clinics/facilities with good access to the proposed patient/subject population. The investigators have been responsive to the most prior critiques and amended the submission with additional data and revisions that strengthen the project. They have added sufficient details about the proposed analysis of DMD data and added an investigator with expertise in this area. However, the panel noted a few minor weaknesses that reduced a bit the overall scientific merit and impact of the application. Weaknesses include lacking rationale for choice of Fitbit and its validity; insufficient justification for not piloting the p3RP alone in Phase 2, rather than only piloting the combined p3RP-DMD; lack of information about the demographics of the musculoskeletal pain conditions that are likely to be referred; lack of details regarding acceptable wear time (e.g. how many hours per day) and thresholds for different levels of physical activity; no mention of whether there is expectation of weight loss due to increased exercise; and some concerns about the complexity of the intervention and the density of the proposal. Overall, this is an excellent proposal with a high level of innovation and significance and a few addressable concerns in the Approach. The committee expresses high enthusiasm for this application that will likely have a high impact in the field of mind-body interventions for patients with chronic musculoskeletal pain.

DESCRIPTION (provided by applicant): Integrating mind body skills with physical activity to improve physical and emotional outcomes in patients with heterogeneous chronic pain This is a new investigator R34 application that follows the recently released IMMPACT criteria, and the International Classification of Functioning, Disability and Health (ICF) framework to adapt a multimodal, theory grounded mind body program (The Relaxation Response Resilience Program; 3RP) with prior success in patients with medical illness in pilot and effectiveness studies, for the needs of patients with chronic pain and to specifically target increased physical activity (p3RP), with the Fitbit, a digital monitoring device (DMD) which provides an objective, real time measurement of increased activity (e.g., number of steps, number of active minutes) with built in reinforcement (p3RP-DMD). Available psychosocial interventions for chronic pain are failing in critical outcomes with effect sizes for physical and emotional functioning that are small and fade over time. Although physical activity is associated with increased health outcomes in patients with chronic illness including pain, physical activity programs are difficult to implement in this population due to barriers associated with motivation, adherence, mood, and ineffective coping skills. The arrival of user friendly DMDs with high adherence represents an opportunity to increase physical and emotional functioning in patients with chronic pain, by incorporating quota based walking within mind body treatments, thus problem solving barriers to

increased activity. DMDs allow both addressing and objectively assessing improvements in function. Our guiding hypothesis is that the synergistic interaction between the evidence-based 3RP program (that includes mindfulness meditation, adaptive thinking and positive psychology skills) which we plan to adapt for the needs of patients with chronic pain including increased physical activity, with the Fitbit DMD to reinforce quota-based pacing and engagement in meaningful daily activities, is the most effective and efficient way to improve both physical (self-report, objective and performance based) and emotional (depression and anxiety) function in chronic pain patients. In a subsequent R01, we plan to test the hypothesis that the p3RP-DMD is superior to the p3RP and to an educational control, the pain specific Health Enhancement Program (pHEP) in improving and sustaining improvements in physical and emotional outcomes. Using the R34 NCCIH mechanism, we propose to use an iterative mixed methods strategy to adapt the 3RP for the specific needs of chronic pain patients including increased activity, and for incorporation of the Fitbit DMD, to maximize their feasibility, acceptability, and credibility. HEP, a dose and time matched educational control developed by our team and adapted for chronic pain (pHEP) is already used in our Pain Clinic and does not require further feasibility testing.

PUBLIC HEALTH RELEVANCE

Chronic pain is prevalent and costly, and current available psychosocial treatments fail to provide sustained improvement in outcomes. Although mild physical activity like walking is associated with improvements in outcomes in patients with chronic illness including pain, current mind body interventions do not directly address or assess improvements in activity. Incorporating small, user friendly and inexpensive DMDs that provide real time reinforcement of activity into mind body interventions represents an opportunity to bolster both physical and emotional function in patients with chronic pain, which is consistent with recommendations from new IMMPACT 2016 criteria for pain trials.

CRITIQUE 1

Significance: 2
Investigator(s): 2
Innovation: 2
Approach: 5
Environment: 2

Overall Impact:

This application describes a multi-phase project in which two self-management interventions to treat chronic pain will be developed, refined and pilot tested. The interventions are based on an established protocol that has been utilized in other populations (3RP). Strengths of the project include the overall concept of integrating physical activity as a treatment target and integrating the Fitbit, the investigative team, and the feasibility of the proposed design. The investigators have made clear links between this proposal and a future larger trial. Successful completion of this project will position the investigators to scale a larger trial. The project as proposed is aligned with the RFA goals.

There are a few minor weaknesses in this proposal. Specifically, the description of the theoretical underpinnings of the intervention and the sheer amount of content seem unfocused and unwieldly, respectively. The intervention and conceptual model need to be streamlined as a result of this project, before scaling a larger trial. That said, the intervention seems to be sufficiently developed to justify the proposal, and refining the intervention is one of the main stated aims.

Methodologically, some minor changes to the design may improve the project. If the main goal of this project is to develop and pilot test two interventions (p3RP and p3RP-DMD), then please consider offering these separately in the open pilot stage, rather than administering only the p3RP-DMD and then asking about the components separately. This phase appears to be the primary foundation for revising these interventions, so dedicating more effort to this appears warranted. Conduct 2 groups,

one for each intervention. Alternatively, if it is that easy to separate the components in qualitative interviews, how integrated are they really?

This reviewer has minor concerns about the ability of the phase 1 focus groups to comment meaningfully on the content. Things that would be reassuring would be explaining that participants in the focus groups would have an opportunity to review all the materials, a priori, and that there would be some discussion of how closely the groups would mirror the manuals. It would also be relevant to have the focus group participants get to try some of the exercises (rather than read them) before commenting.

1. Significance:
Strengths

- The integration of physical activity (PA) is the significant. PA is included as a target for intervention and thoughtfully measured in multiple ways as a primary outcome. The authors clearly describe how the pilot/feasibility studies will lead to the development of a more definitive study. There is sufficient knowledge upon which to base the proposed intervention and design. The proposed project will yield specific information needed to inform a larger trial.
- The integration of the popular and appealing Fitbit is great. The fact that this provides feedback to augment the intervention and is so clearly tied in with the rationale is an additional strength.

Weaknesses

- The proposed interventions are indeed multi-modal - but perhaps too multi-modal to be easily disseminated. Streamlining the interventions and the conceptual model will be necessary to develop an intervention with greater potential impact. As written the current intervention may be too complex to be easily disseminated, which would detract from its' significance. The fact that the investigators will be focusing on intervention development and refinement in this proposal minimizes the impact of this concern. More thought needs to go into the psychological piece of the intervention. The PA component is the star of the show, conceptually and methodologically.

2. Investigator(s):
Strengths

- The psychologist investigators have a deep appreciation for the many theoretical underpinnings/approaches that inform the intervention. It is easy to see the appeal of a multi-modal intervention and appreciate the numerous mechanisms by which this intervention could have a positive impact.
- The team has appropriate qualifications and the inclusion of investigators is sufficiently supported.

Weaknesses

- No major weaknesses noted.

3. Innovation:
Strengths

- Integration of a Fitbit is innovative. This is an appealing and existing technology that is easy to obtain and use. Including a PA expert ensures that this most innovative part of the grant will be captured and optimized.

Weaknesses

- No major weaknesses noted.

4. Approach:
Strengths

- Comprehensive conceptualization of physical function, including of patient reported physical activity, plus objective walking test plus steps. Clearly explained how the IMPACT recommendations informed this selection of PA measures. Measures, theory (IMPACT) and model around physical activity make sense. Clever to use accelerometers as the outcome since they do not provide feedback and can be used equivalently in both conditions.
- Well defined specific aims are tied clearly to necessary steps to prepare for a larger trial.

Weaknesses

- In Aim 1, Phase 2, the authors propose to “pilot and assess the p3RP-DMD intervention in a group of 10 chronic pain patients and conduct exit interviews.” They suggest that “the exit interviews will ask questions targeting the DMD and 3RP separately and integrated, thus gathering specific information for subsequent adaptation of both p3RP and p3RP-DMD in an efficient manner.” This reviewer unclear how these will be administered together but asked about separately in a way that will inform the development of both the p3RP and the p3RP-DMD adaptation. This is not the place for efficiency. Please consider administering 2 open pilots, one for each intervention exactly as you would propose to use it in a larger trial. Conduct exit interviews with participants in each condition to get feedback specific to that condition.
- Conceptual question: the proposal is based on the premise that the 3RP program is more effective than unimodal programs that have been shown to be effective. The support for this statement is questionable. If “unimodal” programs are shown to be effective and are based on, say, 10 sessions of that intervention (e.g., CBT, MBSR), and the proposed intervention combines elements of multiple programs, how do the authors know that the multi-modal combination doesn’t dilute the effectiveness of original programs? The preliminary data on the effectiveness of the 3RP program is noted, but it seems an overstatement to say this is more effective than more established unimodal therapies- was there empirical support for this statement?
- It is not entirely clear how the ICF was integrated. This needs to be better explained.
- There may be too much in the intervention content. For example, how does one cover “yoga/walking meditation” in a fraction of a class? How does one cover LKM in a portion of a session? This seems like a sampling of a lot of techniques without in depth practice of any one skill.
- Authors suggest the intervention is consistent with theoretical models of the fear-avoidance model of pain, cognitive model of pain, ACT, mindfulness, positive psychology. This seems appropriate, yet too unfocused as written. Moving forward, this rationale needs to be simplified and streamlined.
- Unclear how the model in figure 2 illustrates the synergistic influence of the 3PR plus DMD.
- Physical activity measures more thoughtfully articulated than emotional function measures. Unclear whether measures of coping, catastrophizing are outcomes. Unclear how the measures map onto the conceptual model.
- The conceptual pathway from the intervention through the goal of increasing activity, and the associated measures, is well-articulated. This is less true for the psychological goals. It looks like one of the primary objectives is to change attributions/knowledge about pain, and increase skills to invoke a RR and/or more adaptive thoughts in the face of pain. It is unclear how the 7 Relaxation Response Skills and the 5 plus elements of the pain/activity awareness cycle translate to the emotional function outcomes. Unclear that depression and anxiety are the most

obvious ways to assess if psychological goals of the intervention are met. Model does not explain hypothesized relationships. Consider measuring social participation, pain knowledge, use of relaxation strategies - need a parallel to the organized and clean physical function outcomes and rationale.

- Is there homework for those in the p3RP intervention, or only in the p3RP-DMD intervention? Is there an attention match for the daily experience of having a DMD? Are there smart goals for those in the p3RP condition? This is a fundamental ingredient of self-management.

5. Environment:
Strengths

- Information provided suggests that the target sample size can be enrolled in the timeframe. There appear to be sufficient participants available at the proposed site and existing working relationships and physical proximity among the investigators. Infrastructure and resources are available to support the investigators in completing this project.

Weaknesses

- No major weaknesses noted.

Protections for Human Subjects:

Acceptable Risks and/or Adequate Protections

- Inclusion and exclusion criteria are justified and appropriate.
- Unclear if there will be any compensation for participants - this is a time intensive study so I would hope there would be some compensation.

Data and Safety Monitoring Plan (Applicable for Clinical Trials Only):

- Acceptable

Inclusion of Women, Minorities and Children:

- Sex/Gender: Distribution justified scientifically
- Race/Ethnicity: Distribution justified scientifically
- For NIH-Defined Phase III trials, Plans for valid design and analysis: Not applicable
- Inclusion/Exclusion of Children under 18: Excluding ages <18; justified scientifically

Vertebrate Animals:

Not Applicable (No Vertebrate Animals)

Biohazards:

Not Applicable (No Biohazards)

Resubmission:

- Improved application.

Authentication of Key Biological and/or Chemical Resources:

Not Applicable (No Relevant Resources)

Budget and Period of Support:

Recommend as Requested

- Only personnel expenses are included in the budget. Unclear if there is budget for subject renumeration. This is a time-intensive study for participants and some compensation for their time would be expected and appropriate.

Additional Comments to Applicant:

- This reviewer might suggest that investigators lead the first round of pilot groups to get firsthand experience with packing this much material into a session. Even a highly skilled an efficient group leader will have trouble with the amount of material proposed.
- Consider shrinking the phase 3 portion or the study to 2 cohorts, or build in some iterative components. Ultimately a goal of this application should be to refine the intervention, reduce the number of core skills taught/identify the key skills.

CRITIQUE 2

Significance: 1
Investigator(s): 2
Innovation: 1
Approach: 1
Environment: 1

Overall Impact:

This is a revised R34 application. Overall this revision seems to properly address all the issues that were raised in the last cycle. In the last cycle, this application was reviewed very positively and now it seems it has become much better.

1. Significance:
Strengths

- The proposal will examine feasibility of component of a relaxation program combined with actigraphic monitoring and conditioning in chronic pain patients.
- The combination of approaches with objective outcome measure of activity is interesting and certainly worthwhile to explore.
- This application was favorably reviewed in the last cycle.
- The authors carefully address the weaknesses pointed in the last review and as a result this is a much better application.

Weaknesses

- No weaknesses noted.

2. Investigator(s):
Strengths

- Investigative team seems to cover all aspects of the study.

Weaknesses

- No major weaknesses noted.

3. Innovation:
Strengths

- The combination of mind-body intervention with objectively monitored exercise is innovative and has important clinical potential.
- Given that this is an R34 application with emphasis on feasibility and piloting, the concepts presented here are certainly innovative.

Weaknesses

- No weaknesses noted.

4. Approach:
Strengths

- The overall design remains the same as in the last application.
- The authors will test feasibility recruitment for relaxation and relaxation with Fitbit added activity inducement and monitoring.
- The authors carefully address all major points raised by multiple reviewers from the last cycle.

Weaknesses

- No weaknesses noted.

5. Environment:
Strengths

- Excellent

Weaknesses

- No weaknesses noted.

Protections for Human Subjects:

Acceptable Risks and/or Adequate Protections

- Adequate

Data and Safety Monitoring Plan (Applicable for Clinical Trials Only):

  Acceptable
  - Adequate

Inclusion of Women, Minorities and Children:

- Sex/Gender: Distribution justified scientifically
- Race/Ethnicity: Distribution justified scientifically
- For NIH-Defined Phase III trials, Plans for valid design and analysis: Not applicable
- Inclusion/Exclusion of Children under 18: Excluding ages <18; justified scientifically

Vertebrate Animals:

Not Applicable (No Vertebrate Animals)

Resubmission:

- Responsive to prior critique.

Authentication of Key Biological and/or Chemical Resources:

Not Applicable (No Relevant Resources)

Budget and Period of Support:

Recommend as Requested

CRITIQUE 3

Significance: 2
Investigator(s): 3
Innovation: 2
Approach: 3
Environment: 3

Overall Impact:

This is a strong proposal that is likely to make a positive contribution to the field of mind-body interventions for pain. The investigators have been responsive to the reviewer's comments resulting in a well-designed study that is likely to yield important feasibility related outcomes. The investigators have substantial relevant experience and are well suited for the project. There is excellent attention given to critical issues including a strong conceptual model, well-articulated methods for assessing feasibility and identification of key milestones. There are still some weaknesses related to the objective physical activity related measures (Actigraph and Fitbit); greater engagement of the physical activity consultant and more attention to the physical activity research literature could remedy. Overall, the strengths outweigh the weaknesses, and this reviewer has high enthusiasm for this project.

1. Significance:
Strengths

- The proposed project addresses the very prevalent and costly problem of chronic pain, which has become a national priority.
- The applicants do a good job in describing how the results of the proposed work will lead to the development of a larger R01 study.
- Proposed work is well justified; upon completion, important issues (e.g. satisfaction, acceptability, feasibility) will have been addressed.
- The rational for the Relaxation Response Resiliency Program (3RP) is well justified and is based on a solid evidence base of its individual components.

- The study population (chronic pain), intervention (mind-body, with and without real-time physical activity monitoring) and choice of outcome measures align well with NCCIH priorities and recommendations in the pain field (IMMPACT group).

Weaknesses

- No major weaknesses noted.

2. Investigator(s):
Strengths

- Excellent team of investigators with the experience and expertise in clinical research, pain, the interventions and the self-report measures.
- Preliminary research of the intervention (3RP) by the investigators is promising.

Weaknesses

- While the addition of Dr. James as a consultant on physical activity is an improvement over the first submission, there are still issues surrounding the objective physical activity monitoring devices (see below); this suggests greater engagement is required.

3. Innovation:
Strengths

- Use of objective physical activity monitoring devices to simultaneously reinforce/motivate (an important evidence based behavioral strategy) AND collect data; this is a much needed area of exploration in the pain field.
- Combining mind body programs with objective physical activity monitoring devices to improve physical and emotional variables related to chronic pain is an innovative approach which is likely to yield advances in the field.

Weaknesses

- No major weaknesses noted.

4. Approach:
Strengths

- Overall, approach is methodologically rigorous. Excellent conceptual model; very well thought out and reflects what is known about pain and physical activity and how mind body approaches (with and without device monitoring) might improve outcomes.
- Interventions well described and appropriate for end goals.
- Inclusion/exclusion criteria well described and justified. Appreciate recognition that severe mental illness is contraindicated for interventions involving mindfulness/contemplation.
- Qualitative data collection and analysis is rigorous.
- Appropriate choice of quantitative measures.
- Tasks and deliverables well described and necessary for a future R01.
- Investigators appropriately refrain from efficacy analyses.
- Sample size based on feasibility related measures.

Weaknesses

- Appreciate why the investigators chose the Fitbit due to its popularity and user-friendliness; this is important. However, would have liked to see more attention paid to its validity in comparison to other devices. The use of the Actigraph for pre-/post measurement (which is well validated), mitigates this concern somewhat.
- Missing required draft clinical protocol and draft consent forms required in Appendix.
- Limiting focus on walking seems unnecessary and then prevents those with some physical disabilities from participating (note that accelerometer data can be collected in wheelchair bound individuals).
- Lacking information and details regarding acceptable wear time (e.g. how many hours per day); also, thresholds for different levels of physical activity (e.g. light, moderate, etc.).
- Time allowed for data cleaning, especially for the physical activity data is likely insufficient. If more attention is paid in methods to ensuring data collection approaches which will minimize missing data (e.g. having insufficient)

5. Environment:
Strengths

- Excellent scientific environment.
- Letters of support provided.

Weaknesses

- Unclear subjective pool.

Protections for Human Subjects:

Acceptable Risks and/or Adequate Protections

- Human subject's protections acceptable.

Data and Safety Monitoring Plan (Applicable for Clinical Trials Only):

- Acceptable
  - DSMB will be appointed;

Inclusion of Women, Minorities and Children:

- Sex/Gender: Distribution justified scientifically
- Race/Ethnicity: Distribution justified scientifically
- For NIH-Defined Phase III trials, Plans for valid design and analysis: Not applicable
- Inclusion/Exclusion of Children under 18: Excluding ages <18; justified scientifically

Vertebrate Animals:

Not Applicable (No Vertebrate Animals)

Resubmission:

- Initial reviewers' comments addressed.

Authentication of Key Biological and/or Chemical Resources:

Not Applicable (No Relevant Resources)

Budget and Period of Support:

Recommend as Requested

- Suggest greater effort for physical activity consultant and personnel for data cleaning and preparation physical activity data.

CRITIQUE 4

Significance: 2
Investigator(s): 2
Innovation: 1
Approach: 3
Environment: 1

Overall Impact:

This proposed project, which is a revised resubmission of a previous proposal, addresses an important and prevalent public health issue, chronic pain, and examine and compare feasibility of a mind-body skills program adapted for patients with chronic pain (p3RP) with a mind-body skills program that is integrated with use of a Fitbit digital monitoring device and quota-based walking program (p3RPDMD). The project is quite innovative, the investigative team and study environment are outstanding, and the proposal is thoughtfully articulated and responsive to reviewer comments. There are a few minor weaknesses that detract from the proposal, including insufficient justification for not piloting the p3RP alone in Phase 2, rather than only piloting the combined p3RP-DMD (instead, only in phase 3 is the newly adapted p3RP feasibility tested, when it is compared with the p3RP-DMD intervention); lack of clarity regarding how much encouragement of physical activity there is in the p3RP, as compared with the p3RP-DMD; lack of information about the demographics of musculoskeletal pain conditions that are likely to be referred (only fibromyalgia and low-back pain are mentioned); no mention of whether there is expectation of weight loss due to increased exercise, which might be expected particularly in the p3RP-DMD condition. The proposal gives only cursory information in the Approach section regarding the role of patients' physicians in determining their patients' participation in the trial. However, this point is made in the Protection of Human Subjects section, the investigators state that "all referring physicians will be asked to document that there are no medical contraindications to a graded walking program for their patients" and that "participants will be asked if they experienced any negative outcomes because of becoming more physically activity during that day." In spite of these weaknesses, the study will make a valuable contribution to the field in its feasibility testing of a combined mind-body and physical activity intervention for patients with chronic musculoskeletal pain.

Protections for Human Subjects:

- Acceptable

Data and Safety Monitoring Plan (Applicable for Clinical Trials Only):

- Acceptable

Inclusion of Women, Minorities and Children:

- Sex/Gender: Distribution justified scientifically

- Race/Ethnicity: Distribution justified scientifically
- For NIH-Defined Phase III trials, Plans for valid design and analysis: Not applicable
- Inclusion/Exclusion of Children under 18: Excluding ages <18; justified scientifically

Vertebrate Animals:
Not Applicable (No Vertebrate Animals)

Biohazards:
Not Applicable (No Biohazards)

Resubmission:

- Well-responsive to previous critiques.

Authentication of Key Biological and/or Chemical Resources:
Not Applicable (No Relevant Resources)

Budget and Period of Support:
Recommend as Requested

THE FOLLOWING SECTIONS WERE PREPARED BY THE SCIENTIFIC REVIEW OFFICER TO SUMMARIZE THE OUTCOME OF DISCUSSIONS OF THE REVIEW COMMITTEE, OR REVIEWERS' WRITTEN CRITIQUES, ON THE FOLLOWING ISSUES:

PROTECTION OF HUMAN SUBJECTS: ACCEPTABLE

INCLUSION OF WOMEN PLAN: ACCEPTABLE

INCLUSION OF MINORITIES PLAN: ACCEPTABLE

INCLUSION OF CHILDREN PLAN: ACCEPTABLE

COMMITTEE BUDGET RECOMMENDATIONS: The budget was recommended as requested.

Footnotes for 1 R34 AT009356-01A1; PI Name: Vranceanu, Ana-Maria

NIH has modified its policy regarding the receipt of resubmissions (amended applications). See Guide Notice NOT-OD-14-074 at http://grants.nih.gov/grants/guide/notice-files/NOT-OD-14-074.html. The impact/priority score is calculated after discussion of an application by averaging the overall scores (1-9) given by all voting reviewers on the committee and multiplying by 10. The criterion scores are submitted prior to the meeting by the individual reviewers assigned to an application, and are not discussed specifically at the review meeting or calculated into the overall impact score. Some applications also receive a percentile

ranking. For details on the review process, see
http://grants.nih.gov/grants/peer_review_process.htm#scoring.

MEETING ROSTER

The roster for this review meeting is displayed as an aggregated roster that includes reviewers from multiple AT Special Emphasis Panels Meetings
for the 2017/05 council round.
This roster for AT is available at:
http://public.era.nih.gov/pubroster/Reports?DOCTYPE=SEP&DESFORMAT=PDF&AGENDA_SEQ_NUM_P=316981
